# Plasticity of Dispersal‐Related Larval Traits in the Clown Anemonefish *Amphiprion percula*

**DOI:** 10.1002/ece3.71967

**Published:** 2025-08-15

**Authors:** Robin K. Francis, Kurt G. Castro, Sadie Thompson, Isabela Trumble, John E. Majoris, Peter M. Buston

**Affiliations:** ^1^ Department of Biology and Marine Program Boston University Boston Massachusetts USA; ^2^ Department of Biological Sciences University of Southern California Los Angeles California USA; ^3^ Sea Education Association Environmental Studies at Woods Hole Falmouth Massachusetts USA; ^4^ Department of Life Sciences Texas A&M University‐Corpus Christi Corpus Christi Texas USA

**Keywords:** adaptive parental effects, context dependent strategy, phenotypic plasticity, population connectivity

## Abstract

A major goal in marine ecology is to understand patterns of larval dispersal and population connectivity. Dispersal plasticity allows for adaptive variation in dispersal phenotypes in response to variation in environmental conditions and may help to explain intraspecific variation in dispersal distances. However, this phenomenon has only been hypothesized for marine fishes. Here, we test the hypothesis that parents produce larvae with different dispersal‐related traits in response to variation in environmental quality using the orange anemonefish, *Amphiprion percula* . By manipulating food rations in a crossover experimental design, we show that parents produce larger offspring on low‐food rations than on high‐food rations. However, there was no effect of parental diet on larval critical swimming speed. We also show that parents produce larvae with smaller otolith cores while on low‐food rations, which, in combination with parentage analyses, may provide a way to test the dispersal plasticity hypothesis in the field. This study shows that parents can produce different larval phenotypes in response to variation in environmental conditions, demonstrating plasticity in a dispersal‐related larval trait that may help to explain observed variation in *A. percula* larval dispersal distances. Incorporating dispersal plasticity into our understanding of marine dispersal patterns may enhance our understanding of marine metapopulation ecology, fisheries management, and conservation.

## Graphical abstract

In this study, we investigate whether dispersal‐related larval traits are plastic in response to parental habitat quality in a marine fish: the clown anemonefish, *Amphiprion percula*. Results from this study show that parents can produce different larval dispersal‐related phenotypes in response to variation in food rations, which may explain some of the observed variation in *A. percula* larval dispersal distances.

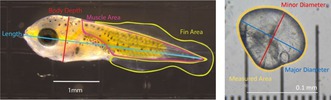

## 1 Introduction

The movement of individuals from their natal habitat to another, or dispersal, is a fundamental driver of population dynamics and species distributions (Botsford et al. 2009; Hastings and Botsford 2006; Clobert et al. 2004). Dispersal distances traveled by individuals within a species are often highly variable (Bowler and Benton 2005). There are many reasons why individual dispersal distances may vary: passive dispersers may reflect the natural variation in environmental drivers (e.g., wind dispersing seeds; Kuparinen 2006), while active dispersers may reflect adaptive behaviors in response to environmental or social cues (e.g., resource depletion, Baguette et al. 2011; or overpopulation, Sutherland 1969). Currently, the causes of intraspecific variation in dispersal distances for marine fishes are unknown. As one of the largest and most ecologically and economically significant taxa of organisms, understanding the fundamental drivers of marine fish population dynamics is critical.

Many marine fishes disperse from their natal habitat during the larval stage (Cowen et al. 2007). One useful way to depict the spatial pattern of larval dispersal is using dispersal kernels (Nathan and Muller‐Landau 2000). A dispersal kernel is a probability density function that provides the probability of successful dispersal over a given distance (e.g., D'Aloia et al. 2015). From the six fish species that have had their larval dispersal kernel empirically measured, there appears to be substantial intraspecific variation in dispersal distances among individuals ( *Elacatinus lori* , D'Aloia et al. 2015; *Plectropomus leopardus, Plectropomus maculatus*, Williamson et al. 2016; *Amphiprion percula* , *Chaetodon vagabundus* , Almany et al. 2017; *Amphiprion clarkii* , Catalano et al. 2021). Individual larval behavior and morphological traits may be potential causes of this variation (Johnson et al. 2010; Nanninga and Manica 2018; Faillettaz et al. 2018; Burgess et al. 2022; Wu and Seebacher 2022). Marine fish larvae are active dispersers with extraordinary swimming abilities allowing them to outpace local reef currents (Fisher et al. 2005) and with early developmental onset of sensory abilities allowing them to navigate toward suitable habitat (Majoris et al. 2021). Comparisons between multiple marine fish species have shown correlations between larval traits, such as swimming speed and larval body length (Fisher et al. 2005; Majoris et al. 2019), with indicators of population connectivity and dispersal distance (Nanninga and Manica 2018; Majoris et al. 2019). High‐resolution biophysical modeling has also linked variation in larval traits, such as swimming speed, to variation in larval dispersal kernels (Burgess et al. 2022). Together, these studies suggest that variation in dispersal distance may be explained by variation in larval dispersal‐related traits.

Adaptive plasticity of larval traits is a plausible cause of intraspecific variation in individual dispersal distances. This type of phenotypic plasticity is widespread because a context‐dependent dispersal strategy will often have an evolutionary advantage over a fixed phenotype (Bowler and Benton 2005; Ronce 2007; Arendt 2015). Since marine fish dispersal occurs during the larval stage just after hatching, it is likely that marine fish dispersal phenotypes are responsive to cues from the parental environment (e.g., Cortese et al. 2022). Such adaptive parental effects on offspring dispersal distance have been described several times in terrestrial systems (Benard and McCauley 2008; Clobert et al. 2009). For example, parents may respond to predator occurrence (e.g., aphids; Weisser et al. 1999), parasitic load (e.g., lizards; Sorci et al. 1994), nutrient depletion (e.g., plants; Imbert and Ronce 2001), or food availability (e.g., termites; Korb and Katrantzis 2004) by altering offspring traits associated with dispersal distance. These phenotypic changes result in offspring that are either equipped to disperse far from, or remain close to, their natal habitat, depending on which strategy confers a selective advantage. Despite evidence across terrestrial plants, insects, and vertebrates, dispersal plasticity has only been hypothesized in marine fishes (Nanninga and Berumen 2014; Francis et al. 2024; Cortese et al. 2024).

In this study, we investigate whether dispersal‐related larval traits are plastic in response to parental habitat quality in a marine fish: the clown anemonefish, *A. percula* . Clown anemonefish have become a model system for studying marine fish larval dispersal (Jones et al. 2022), and individual dispersal distances vary over several orders of magnitude (Buston et al. 2012; Almany et al. 2017). Prior evidence supports the hypothesis that dispersal plasticity might underlie this variation in dispersal distance in *A. percula* . Phenotypic plasticity is expected to evolve if individuals (i) experience variation in environmental conditions, (ii) can reliably assess those environmental conditions, (iii) can express different phenotypes in response to environmental conditions, and (iv) if one phenotype has higher relative fitness than the other in one environmental context, and vice versa. In *A. percula* , (i) parents reside in anemones of varying quality (Buston and Elith 2011; Barbasch et al. 2020; Salles et al. 2020); (ii) parents can reliably predict the habitat quality their offspring will encounter due to spatial autocorrelation of multiple habitat quality indicators (Francis et al. 2024; Marrot et al. 2025); and therefore (iv) different dispersal phenotypes will result in different fitness in different environmental contexts (Buston and Elith 2011; Barbasch et al. 2020; Salles et al. 2020). It remains to be shown whether (iii) parents can produce offspring with different larval phenotypes in response to variation in environmental conditions.

Here, we test the hypothesis that parents in poor‐quality environments will produce larvae with traits that will enable them to disperse farther than larvae from parents in high‐quality environments. Using a lab population of *A. percula* , we manipulated parental food rations and measured the effect on critical swimming speed and body size of their offspring. Manipulations of food availability have produced plastic responses in *A. percula* reproduction and parental care both in the lab (Barbasch and Buston 2018) and in the field (Barbasch et al. 2020). We predict that in response to a low‐food ration (i.e., being in a low‐quality environment), parents will produce larger larvae with better swimming abilities, enabling them to disperse farther in search of higher‐quality environments. Lastly, to provide a way to test the dispersal plasticity hypothesis in the field during future studies, we investigate whether larval otoliths (i.e., ear stones) are markers of parental environment quality by measuring otolith core size in response to parental food availability. Results from this study show that parents can produce different larval phenotypes in response to variation in environmental conditions, demonstrating plasticity in dispersal‐related larval traits, which may explain some of the observed variation in *A. percula* larval dispersal distances.

## 2 Methods

### 2.1 Laboratory Population

This study was conducted in the Buston Lab at Boston University from March 2020 to July 2021. The Buston Lab has housed 60 pairs of *A. percula* , originally wild‐caught from Papua New Guinea (supplied by Quality Marine), for the past 10 years. Individuals in the laboratory population were collected while under 30 mm standard length (SL), ensuring they were nonbreeders and their removal would have no impact on the reproductive output of breeders or local population growth (e.g., Buston 2004; Buston and Elith 2011). These now adult fish are housed as breeding pairs: one female and one male (Female SL mean ± standard deviation [SD] 5.32 ± 0.17 cm; male SL 4.02 ± 0.20 cm). We measured the female standard length to the nearest 0.1 cm using calipers at the beginning and the end of the study. These breeding females were growing very slowly (mean change in SL ± SD = 0.14 ± 0.22 cm over the study period). Thus, any observed changes in reproductive output seen in the results can be attributed to changes in the environment.

Pairs are kept in 60 120‐L tanks with unique Tank ID's, connected to an automated, circulating seawater system that maintains conditions of 33–35 ppt salinity, 27°C–29°C, and pH 8–8.3. Water quality testing and aquaria maintenance are conducted per Institutional Animal Care and Use protocol (IACUC protocol #17‐001; see Schmiege et al. 2017; Barbasch and Buston 2018 for detailed animal care methods). Each tank contains sand along the bottom, a terracotta tile for the fish to lay eggs on, and a rock that bears anemones, *Entacmaea quadricolor*. At the start of the experiment, 34 of the tanks had consistent and actively breeding pairs and were selected for manipulation.

### 2.2 Experimental Design

To manipulate the parental environment, we simulated high‐ and low‐quality conditions with two variations of food ration (Barbasch and Buston 2018; Barbasch et al. 2020). Fish were fed food pellets (Piscine Energetics PE pellets, 1 mm slow sinking, manufactured by Mysis) mixed with seawater and a rotating schedule of supplements and vitamins, dispensed from a pipette dropper 6 days a week. For a high‐quality environment, we provided a “high ration” of pellets (0.15 ± 0.006 g). For a low‐quality environment, we provided a “low ration” of pellets (0.05 ± 0.005 g).

The 34 breeding pairs were randomly split into two treatment groups. Half were first placed on the “high ration” treatment (*n* = 17 pairs), and the other half were placed on the “low ration” treatment (*n* = 17 pairs). While pairs reproduce on a lunar cycle, we did not collect offspring for measurements until at least four months to allow the fish to acclimate to their new diet and ensure that they responded to the treatment. Note that it has previously taken just 1 month of food manipulation to affect *A. percula* reproduction (Barbasch et al. 2020). After four months, we collected and measured offspring from each pair over a 3‐month sampling period. The fish received the “first round” of food ration treatment for a total of seven months, and then we switched the treatments.

Pairs that had first received the “high ration” were switched to the “low ration” and vice versa. Pairs that did not reproduce, or from whom we had failed to collect larvae, were removed from the experiment. After another 4‐month acclimation period, offspring were again collected and measured from each pair over the following three months. Collections were discontinued after three months and any pairs that had not reproduced during the “second round” were excluded from the final analyses. The final sample size included 9 breeding pairs that experienced “high ration” then “low ration” and 8 breeding pairs that experienced “low ration” then “high ration”. We successfully sampled and measured 15 larvae from each of the final 17 pairs on both rations (*n* = 510 larvae). This experimental design is powerful because it enables us to compare larvae from the same parents on both diets, controlling for genetic effects (Schlatter et al. 2022) while focusing on effects of the parental environment. Additionally, the order of the feeding treatment is reversed for the two groups to control for the effect of the order of treatment (“first round” or “second round”), and seasonal effects.

### 2.3 Larval Collection and Rearing

Over the entire 14 months, we surveyed the breeding pairs daily and recorded the presence and age of egg clutches. During the sampling period, we sampled one clutch per pair per treatment. On the night prior to expected hatching, a tile with an individual egg clutch was photographed with an underwater camera (Olympus TG‐870). In the field, eggs hatch by day 8 (Buston 2004; Barbasch et al. 2020); however, this varies between individual breeding pairs in the lab, so we estimate the hatch day based on the pairs' previous clutches (7–11 days; 9.25 ± 1.00 days). To ensure our “high ration” and “low ration” treatments successfully induced a “high quality” and “low quality” environment, we tested the effect of treatment on reproduction. We averaged the number of clutches laid per month (i.e., one reproductive cycle; Seymour et al. 2018) during the sampling period and counted the number of eggs in one sample clutch from photographs in ImageJ (version 1.47; Rasband 1997).

After being photographed, we transferred the tile with the egg clutch to a separate 120‐L rearing tank. Each tank had 50 L of water from the parental tank, 50 L of pre‐made saltwater, and 15 rotifers (*Brachionus rotundiformis*) per mL of water to feed the larvae once they hatch overnight. Water quality of the rearing tank was tested and maintained to be the same as the adult tanks (salinity of 33–35 ppt, temperature of 27°C–28°C, pH of 8.0–8.3). To keep the eggs oxygenated without their parents to tend them, air was bubbled over the eggs using an aquarium air compressor, tubing, and an air stone. The morning after hatching, we replenished the rearing tanks with rotifers and subsequently collected 15 live individual larvae for measurements.

### 2.4 Larval Trait Measurements

**FIGURE 1:**
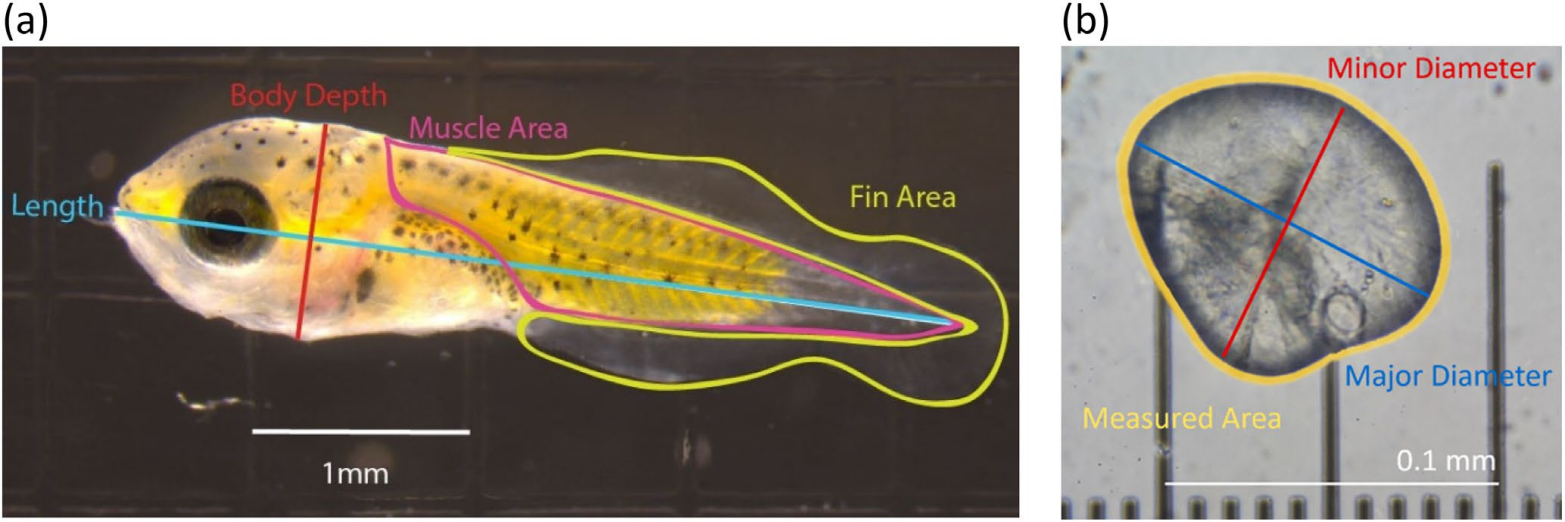
(a) Larval body morphology metrics and (b) otolith metrics. (a) Pictured is a Day 0 *Amphiprion percula* larva. Length is illustrated in blue, body depth in red, muscle area in purple, and fin area in yellow outlines and labels. (b) Pictured is a larval otolith. Major diameter is illustrated in blue, minor diameter in red, and measured area in yellow.

To measure critical swimming speed, or Ucrit (cm/s), a single larva is placed in a swimming flume where it swims against a controlled current speed that is incrementally increased by 2 cm/s every 2 min until the larva fails to maintain its position within the flume's central chamber (see Majoris et al. 2019; modified from the design by Stobutzki and Bellwood 1997). Ucrit is calculated using the following equation:

$$\text{Ucrit}=U+\left(t/t_{i}\times U_{i}\right)$$

where *U* is the penultimate speed, *t* is the time spent at the final speed, *t*_*i*_ is the time increment, and *U*_*i*_ is the speed increment (Brett 1964). The larva is then euthanized with sodium bicarbonate buffered MS‐22, soaked in 10% formalin for 5 min to increase the opacity of the fins, and photographed with a dissecting scope on a 1 mm graticule slide. Images were captured with a Canon 60D digital SLR camera operated with Helicon Remote version 3.9.10 W (Helicon Soft Ltd. 2000). From photos, we measured standard length (length), body depth (depth), muscle body area (muscle area), and total fin area (fin area) using ImageJ (Figure 1a). Each measurement was made in triplicate and averaged for each individual larva.

Length for Day 0 larvae is measured as notochord length (Roux et al. 2019) from the anterior tip of the lower jaw to the posterior end of the notochord. Body depth is measured from the dorsal crest to the ventral side, behind the eye and before the opercle. Muscle area is measured by tracing the visible musculature that starts just behind the head and excludes the gut. Fin area is measured by tracing the extent of the fins. At this age, *A. percula* fins are fused into a single larval fin fold before they develop into the separate dorsal, caudal, and anal fins (Yasir and Qin 2007).

To measure otolith core size, 15 additional larvae were collected from either the same clutch or another clutch from the same parents during the same sampling period. We successfully sampled otoliths from an additional 12–15 larvae from 14 pairs on both rations (*n* = 414 larvae). These larvae were also euthanized and photographed as previously described. One sagittal otolith was dissected from each larva and photographed with a compound microscope on a 0.01 mm graticule slide. The otolith core is visible in older *A. percula* otoliths by a distinct hatch mark (Berumen et al. 2010) followed by daily incremental growth rings (Raventós and Macpherson 2001). However, the morning after hatching, the otolith is just the core with no growth rings, so the entire extent was measured. The cores were not perfectly symmetrical ovals; therefore, we collected multiple measurements. From photos, we measured the otolith's major diameter, minor diameter, and area in ImageJ (Figure 1b). Each measurement was made in triplicate and averaged for each individual larva. We also estimated the otolith core's area from the average major and minor diameter using the equation for the area of an ellipse.

### 2.5 Statistical Analyses

Statistical analyses were conducted in R (R Core Team 2022, R Version 4.2.2). To determine the effect of parental environment on various metrics, we performed mixed models with treatment (two categories) as the predictor variable. We also included round (two categories) to account for differences in offspring produced in the “first round” versus the “second round” of the study period (seasonal effects), and the interaction between treatment and round to account for differences in receiving the “high ration” or the “low ration” during the “first round” or “second round” (i.e., order effects). We included the “tank ID” of the parents as a random effect to control for the lack of independence of larvae from the same parental pair. We removed variables from the model in a backwards stepwise fashion if they were not statistically significant (alpha 0.1), while blocking for treatment, our variable of interest, and keeping the random effect of “tank ID”.

Our first step was to determine whether our treatments reflect high‐quality and low‐quality environments in the wild, eliciting the changes in reproductive output seen in the wild that might also produce an adaptive response in dispersal‐related traits. To ensure our food rations impacted our breeding anemonefish in the way we hoped, we tested whether treatment influenced reproduction. We used the average “number of clutches” laid per month over the sampling period and the “number of eggs” per clutch sampled as response variables.

To determine the effect of treatment on dispersal‐related larval traits, we used larval “Ucrit”, “length”, “depth”, “muscle area”, “fin area”, and otolith “major diameter”, “minor diameter”, “measured area”, and “calculated area” as the response variables. All models were fit using the *lme4* package (Bates et al. 2009). Significance of variables was tested using the *lmerTest* package (Kuznetsova et al. 2020). Except for “number of eggs”, which is a discrete count, we performed a generalized linear mixed model with a Poisson family distribution. Assumptions of normality of residuals and heteroscedasticity were assessed visually using diagnostic plots. Results are presented as reaction norm plots to visually assess the presence of phenotypic plasticity (Schlichting and Pigliucci 1998).

Most importantly, we had a very clear a priori hypothesis that we wanted to test with a carefully designed lab experiment and tailored statistics. Specifically, that there will be an effect of experimental treatment on larval traits, which might be influenced by round or the interaction between treatment and round. We could have measured and included all sorts of additional covariates, e.g., anemone size, female body size, male body size, clutch size, and parental care; the list goes on, but this is not the focus of the study and not generally needed for well‐designed, carefully controlled lab experiments.

## 3 Results

### 3.1 Effect of Treatment on Reproduction

**FIGURE 2:**
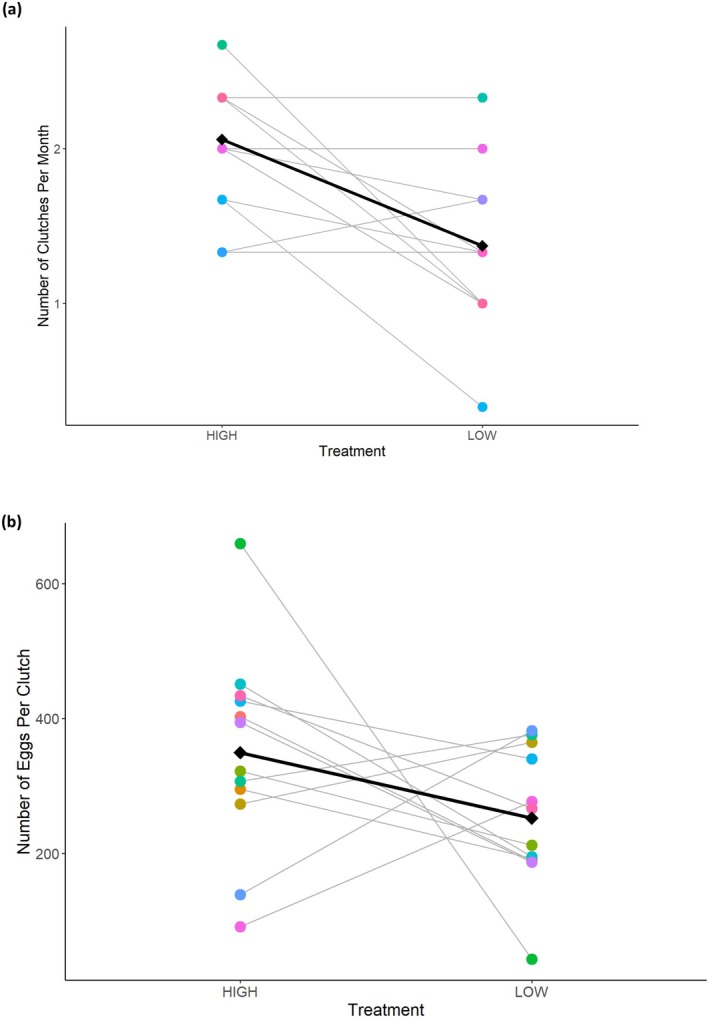
Individual pairs' reaction norms of (a) average number of clutches laid per month and (b) number of eggs per clutch. Each point represents the metric value, per pair, per treatment. Points are individually colored by the unique “tank ID” of each parental pair and may be stacked on top of one another. The gray fitted line connects the pair's measured values and represents the response to the two treatments. Diamonds represent the mean value for all pairs per treatment, and the black line represents the mean response.

While on the “high ration” treatment, pairs laid up to 3 egg clutches per month (mean ± standard error [SE] of 2.06 ± 0.02 clutches), which is the maximum number of clutches observed in the field (Buston and Elith 2011) as well as the maximum number of clutches possible as a result of plentiful resource availability in the lab (Seymour et al. 2018). The “low ration” treatment was severe enough to lower the number of clutches laid per month to 0–2 egg clutches per month (1.37 ± 0.03 clutches), while still enabling some reproduction for the sampling of offspring. The interaction between treatment and round was not a significant predictor and was removed from the final model. However, the round had a significant effect on the number of clutches (“second round”: *t* = 6.76, df = 491, *p* < 0.001). Later in the experiment, during the “second round” of treatment, regardless of treatment, fish laid 0.28 clutches more per month. Treatment had a significant effect on the average number of clutches laid per month (“low ration”: *t* = −26.37, df = 491, *p* < 0.001). Visually assessing individual pairs' reaction norm reveals a pattern of a general population‐level response from one treatment to the next, indicating the presence of plasticity: on average, pairs laid 0.7 clutches less per month while on the “low ration” (Figure 2a).

Pairs responded to the two treatments in a similar way as in a previous study that found plasticity in the number of eggs produced per clutch in response to food availability manipulation in the field (Barbasch et al. 2020). While on the “high ration”, pairs produced clutches with a mean of 316.9 ± 9.65 eggs. While on the “low ration”, pairs produced clutches with 248.4 ± 6.78 eggs. The interaction between treatment and round was not a significant predictor and was removed from the final model. However, round had a significant effect on the number of eggs (“second round” *z* = 79.68, *p* < 0.001). Later in the experiment, during the “second round” of treatment, regardless of treatment, fish laid 151.18 more eggs per clutch. Treatment had a significant effect on the number of eggs per sampled clutch (“low ration”: *z* = −49.87, *p* < 0.001). Visually assessing individual pairs' reaction norm reveals a pattern of a general population‐level response from one treatment to the next, indicating the presence of plasticity: on average, pairs laid an average of 97.3 fewer eggs while on the “low ration” (Figure 2b).

Since the effect of treatment was significant for both the number of clutches laid per month and the number of eggs laid per clutch, we are satisfied that our two feeding rations enabled us to generate the naturally occurring range of variation in *A. percula* reproduction caused by natural variation in environmental quality.

### 3.2 Effect of Treatment on Length

**FIGURE 3:**
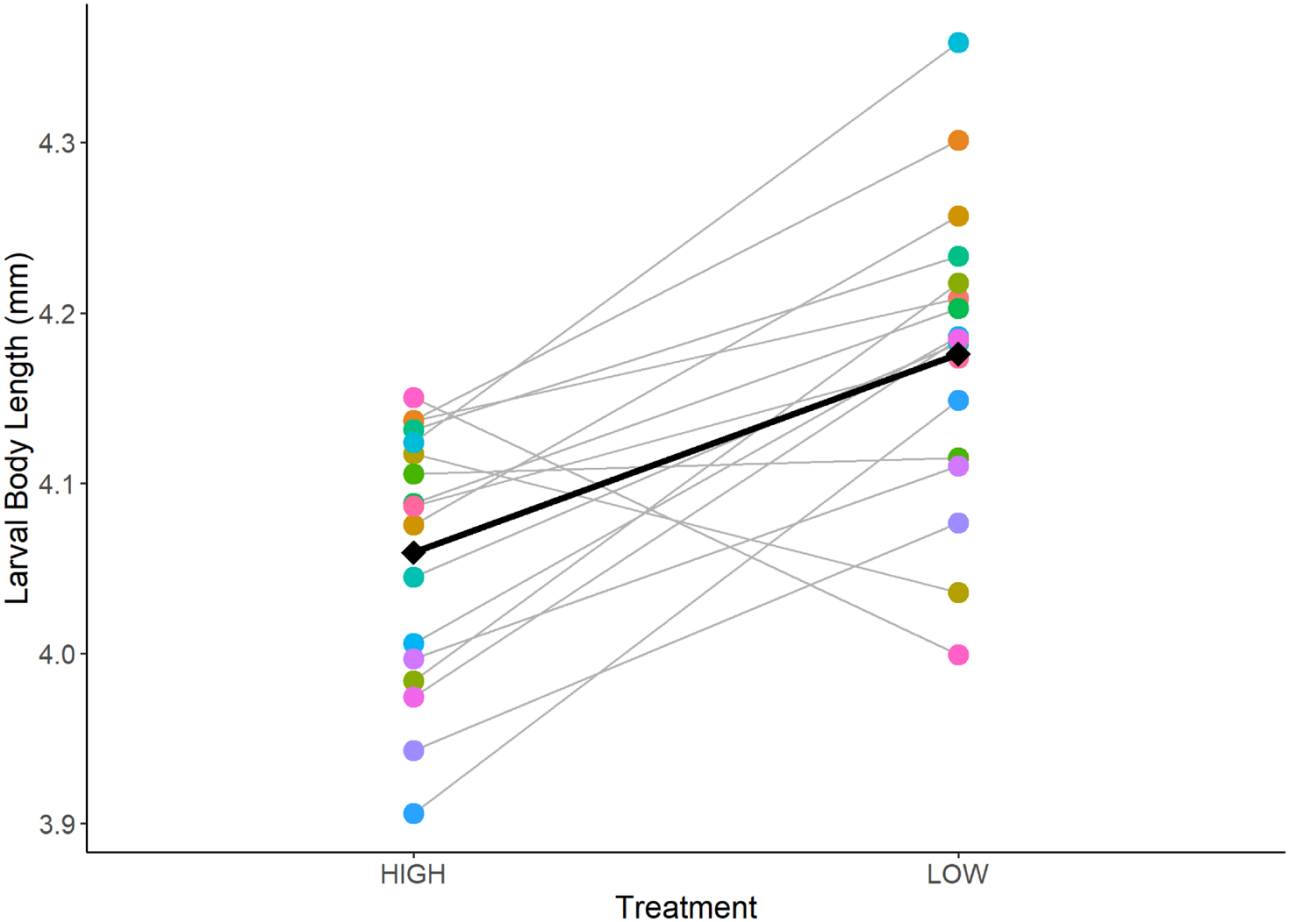
Individual pairs' reaction norms for the length of larvae produced. Each point represents the mean length of larvae sampled, per pair, per treatment. Points are individually colored by the unique “tank ID” of each parental pair and may be stacked on top of one another. The gray fitted line connects the pair's offspring means and represents the response to the two treatments. Diamonds represent the mean length of larvae for all pairs per treatment, and the black line represents the mean response.

Larvae from parents fed the “high ration” had a mean length of 4.06 ± 0.01 mm. Larvae from parents fed the “low ration” had a mean length of 4.18 ± 0.01 mm. Neither round nor the interaction between treatment and round was a significant predictor and was removed from the final model. Results from our mixed model show that treatment had a significant effect on length (“low ration”: *t* = 11.23, df = 492, *p* < 0.001). Visually assessing individual pairs' reaction norm reveals a pattern of a general population level response from one treatment to the next, indicating the presence of plasticity. Larvae were 0.12 mm longer (3% longer) from parents on the “low ration” (Figure 3). There is variation among individuals in the magnitude and slope of the reaction norm. Treatment explains 16% of the variation in length (conditional *R* ^2^ = 0.339, marginal *R* ^2^ = 0.164).

**FIGURE A1:**
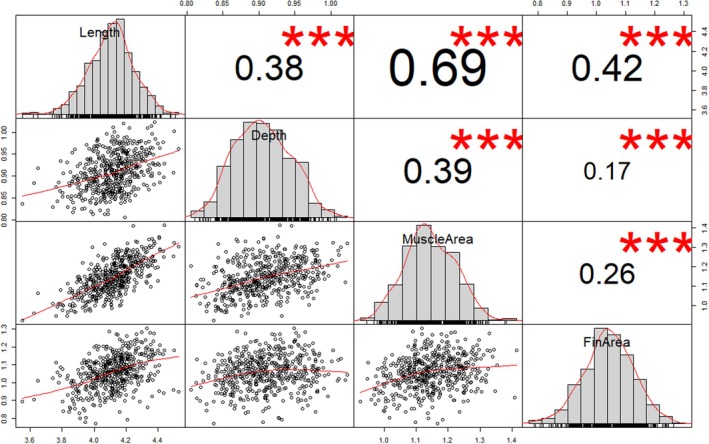
Correlogram of larval morphology. Each row is a body morphology metric, from the top to bottom: larval body length (length), body depth (depth), muscle area (muscle area), and propulsive fin area (Fin Area). The frequency distribution of each variable is shown on the diagonal, with the density curve in red. On the bottom of the diagonal is the bivariate scatterplot with the fitted line in red. On the top of the diagonal is the correlation coefficient and associated significance level (***p < 0.001). The relative font size signifies value of the correlation.

**FIGURE A2:**
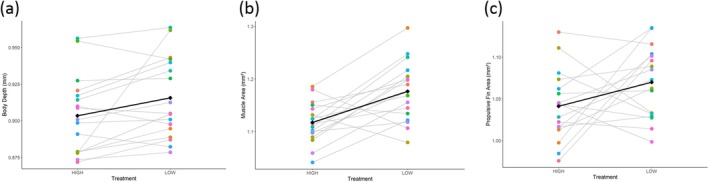
Individual pair reaction norms for (a) body depth, (b) muscle area, and (c) fin area. Each point represents the average metric value for each clutch per pair, per treatment. Points are individually colored by the unique “tank ID” of each parental pair and may be stacked on top of one another. The grey fitted line connects the pair's measured values and represents the response to the two treatments. Diamonds represent the mean value for all pairs per treatment and the black line represents the mean response.

Larval length, depth, muscle area, and fin area were all significantly and positively correlated (Figure A1). For brevity, we only report results for length here. The other three body morphology metrics' final models yielded similar results, showing treatment had a significant effect and that larvae were all‐around larger when parents were on the “low ration” ( Table A1; Figure A2).

### 3.3 Effect of Treatment on Critical Swimming Speed

**FIGURE 4:**
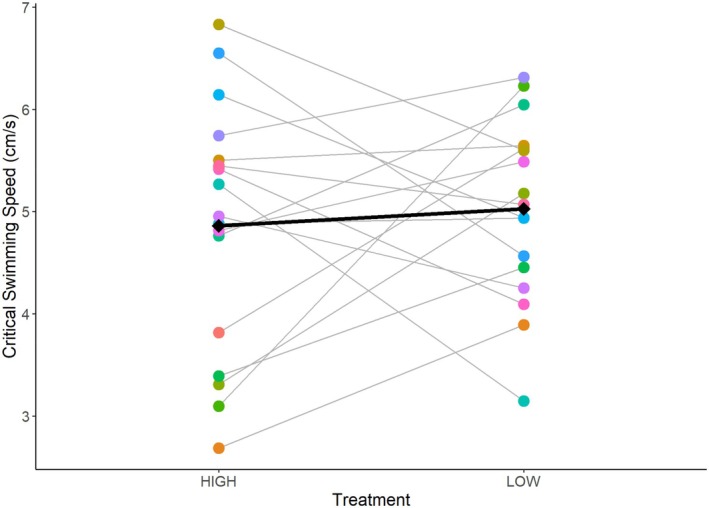
Individual pairs' reaction norms for critical swimming speed (Ucrit) of larvae produced. Each point represents the mean Ucrit of each clutch sampled, per pair, per treatment. Points are individually colored by the unique “tank ID” of each parental pair and may be stacked on top of one another. The gray fitted line connects the pair's offspring means and represents the response to the two treatments. Diamonds represent the mean Ucrit of larvae for all pairs per treatment, and the black line represents the mean response.

Larvae from parents fed the “high ration” treatment had a mean Ucrit of 4.86 ± 0.15 cm/s. Larvae from parents fed the “low ration” treatment had a mean Ucrit of 5.03 ± 0.13 cm/s. The interaction between treatment and round was not a significant predictor and was removed from the final model. Results from our mixed model show that treatment did not have a significant effect on Ucrit (“low ration”: *t* = 1.182, df = 491, *p* = 0.238). Round did have a significant effect (“second round”: *t* = −4.42, df = 491, *p* < 0.001). Larvae swam 0.8 cm/s faster on average during the “second round”, regardless of treatment. However, treatment and round only describe 4% of the variation in larval Ucrit (conditional *R* ^2^ = 0.128, marginal *R* ^2^ = 0.035). Visually assessing individual pairs' reaction norm of mean offspring's Ucrit shows a lack of any general pattern of a population‐level response from one treatment to the next (Figure 4).

### 3.4 Effect of Treatment on Otolith Core Size

**FIGURE 5:**
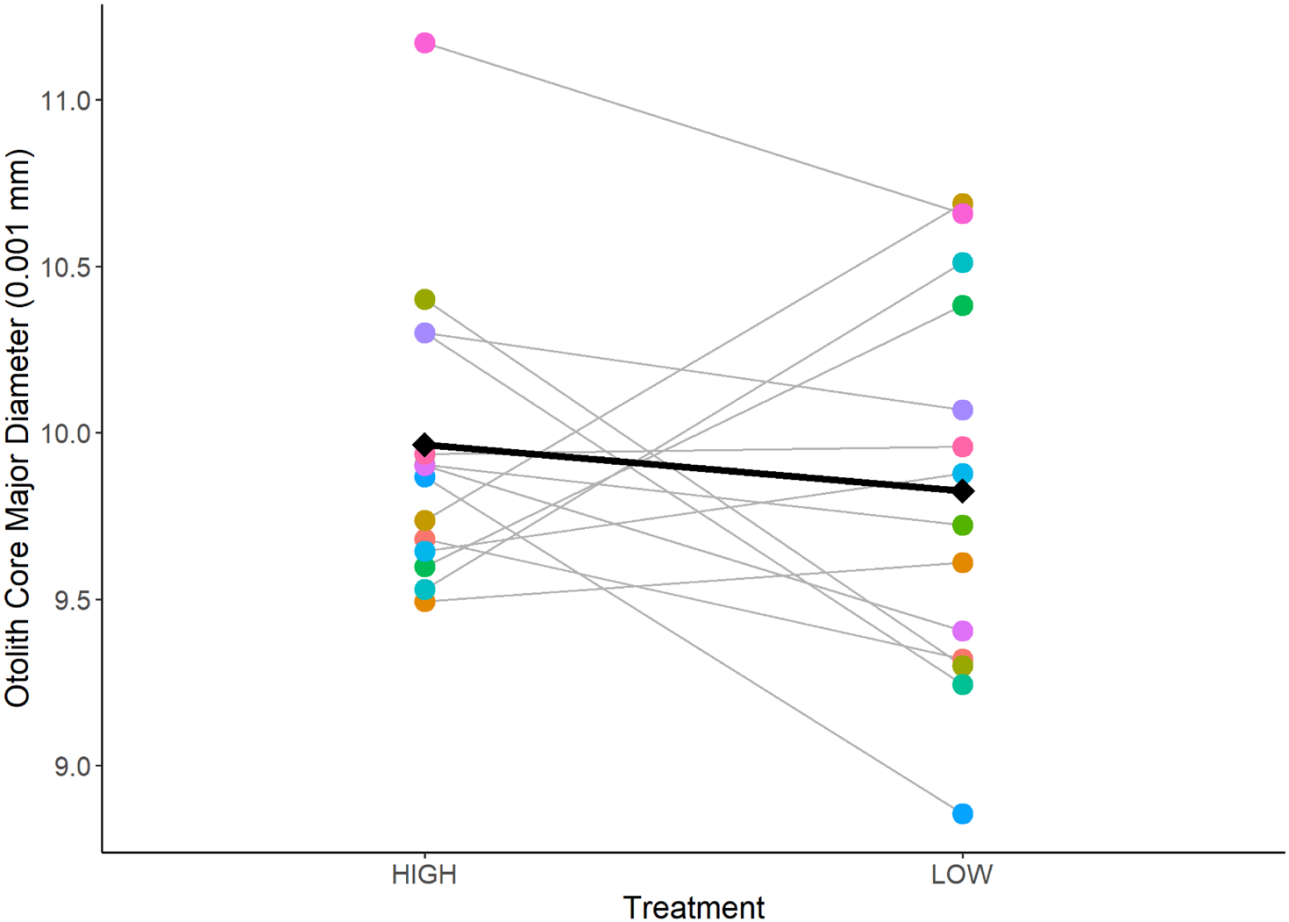
Individual pairs' reaction norms for otolith core major diameter of larvae produced. Each point represents the mean otolith core major diameter of each clutch sampled, per pair, per treatment. Points are individually colored by the unique “tank ID” of each parental pair and may be stacked on top of one another. The gray fitted line connects the pair's offspring means and represents the response to the two treatments. Diamonds represent the mean major diameter of larval otolith cores for all pairs per treatment, and the black line represents the mean response.

Larvae from parents fed the “high ration” treatment had otoliths with a mean ± standard error (SE) major diameter of 99.6 ± 0.4 μm. Larvae from parents fed the “low ration” treatment had otoliths with a mean major diameter of 98.3 ± 0.05 μm. Neither round nor the interaction between treatment and round were significant predictors of otolith size and were removed from the final model. Results from our mixed model show that treatment did have a significant effect (*t* = −2.22, df = 399, *p* = 0.027). The major diameter of larval otoliths was 1.3 μm smaller from parents that were on the “low ration” (Figure 5). There is variation in the magnitude and slope of the reaction norm. However, our model explains less than 0.01% of the variation in otolith size (conditional *R* ^2^ = 0.287, marginal *R* ^2^ = 0.009).

**FIGURE A3:**
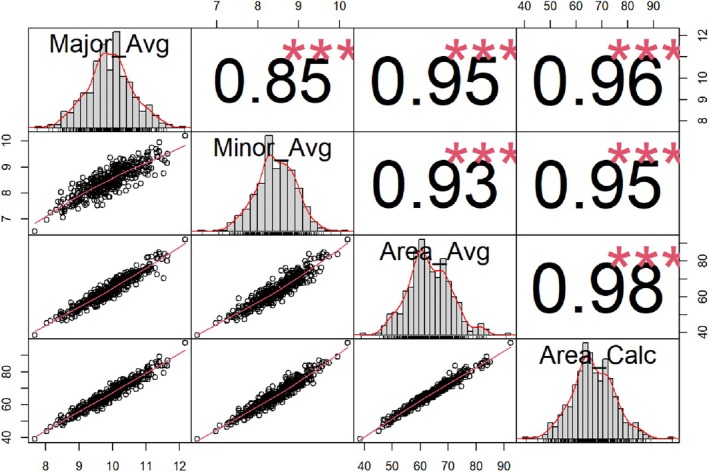
Correlogram of larval otolith metrics. Each row is an otolith metric, from the top to bottom: major diameter (Major Avg), minor diameter (Minor Avg), measured area (Area Avg) and calculated area (Area Calc). The frequency distribution of each variable is shown on the diagonal, with the density curve in red. On the bottom of the diagonal is the bivariate scatterplot with the fitted line in red. On the top of the diagonal is the correlation coefficient and associated significance level (***p < 0.001). The font size signifies value of the correlation.

**FIGURE A4:**
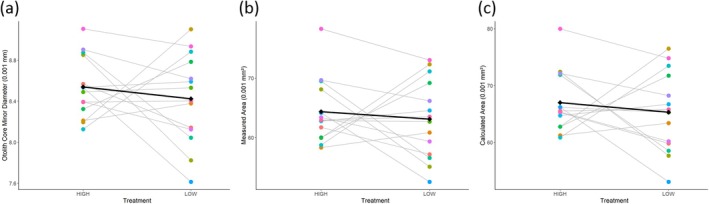
Individual pair reaction norms for otolith (a) minor diameter, (b) measured area, and (c) calculated area. Each point represents the average metric value for each clutch per pair, per treatment. Points are individually colored by the unique “tank ID” of each parental pair and may be stacked on top of one another. The grey fitted line connects the pair's measured values and represents the response to the two treatments. Diamonds represent the mean value for all pairs per treatment and the black line represents the mean response.

Otolith core major diameter, minor diameter, measured area, and calculated area were all significantly and positively correlated (Figure A3). For brevity, we only report results for major diameter here. The other three otolith metrics' final models yielded similar results, showing treatment had a significant effect on the minor diameter and a marginally significant effect on the measured and calculated area (Table A2). Otoliths were all‐around smaller when parents were on the “low ration” (Figure A4).

## 4 Discussion

A major goal in marine population ecology is to determine the causes of variation in larval dispersal distances and consequential population connectivity (Cowen 2002; Cowen and Sponaugle 2009; Buston and D'Aloia 2013). Dispersal plasticity allows for adaptive variation in dispersal phenotypes in response to variation in environmental conditions (Bowler and Benton 2005; Ronce 2007; Arendt 2015) and may help to explain some of the substantial intraspecific variation we observe in marine fish dispersal distances. For the many demersal spawning reef fishes that provide parental care, it is likely that a plastic response in larval dispersal phenotype would be informed by the parental environment. In this study, we experimentally investigated one of the conditions that must be met for plasticity to occur as an adaptive parental effect: whether parents can produce offspring with different phenotypes in response to variation in the parental environment. By manipulating food availability for *A. percula* parents, we created variation in reproductive output consistent with the variation seen in high‐quality and low‐quality habitats in the field (Buston and Elith 2011; Barbasch et al. 2020). By comparing offspring from the same parents while on a “high” and a “low” food ration, we show a plastic response in body size of *A. percula* larvae. Larvae were larger when parents were given the “low” food ration. This result supports the hypothesis that parents in poor quality environments might be producing larvae that can disperse farther, since dispersal ability and body size are positively related in many taxa (Bowler and Benton 2005; Benard and McCauley 2008, and Clobert et al. 2009).

### 4.1 Evidence of Plasticity in Larval Body Size

We might expect parents to produce larger offspring when given greater amounts of food (e.g., Donelson et al. 2009), perhaps because they have more resources to allocate to gametes as yolk reserves (Valdebenito et al. 2015). Alternatively, selection might favor parents that produce larger offspring when provided with less food, so that the larvae are equipped to disperse out of the poor environment. In accordance with our dispersal plasticity hypothesis, we found parents produced larger larvae during the “low ration” than the “high ration” treatment. There is variation in the magnitude and slope of the reaction norm, which might reflect variation in parental state or underlying genetic variation on which selection could act. Our otherwise counterintuitive result is not anomalous; in a study that used a similar experimental design, Reznick and Yang (1993) found female guppies, *Poecilia reticulata* , produced offspring with greater mass and fat reserves when given lower food rations than females given higher food rations. Such responses to poor diets, in which mothers produce larger eggs (e.g., *Drosophila melanogaster* , Vijendravarma et al. 2010; *Daphnia pulicaria* and *Daphnia hyalina* , Gliwicz and Guisande 1992) and/or larvae (e.g., *Armadillidium vulgare* , Brody and Lawlor 1984), have been attributed to adaptive parental effects that may bolster resiliency of offspring born into poor environments (Badyaev and Uller 2009).

Larger body sizes resulting from a poor parental environment may allow offspring to disperse greater distances and have a higher probability of settling in a better environment. Dispersal is a costly life‐history trait (Bonte et al. 2012), and many organisms evolved to have condition‐dependent dispersal‐related traits that result in non‐random dispersal syndromes and preferential survival of certain dispersing individuals (reviewed by Bowler and Benton 2005; Benard and McCauley 2008, and Clobert et al. 2009). Body size is a widespread dispersal‐related trait (Bowler and Benton 2005; Benard and McCauley 2008, and Clobert et al. 2009). In studies of intraspecific variation in natal dispersal, generally larger individuals disperse from their natal habitat more than smaller individuals (e.g., *Enallagma boreale* , Anholt 1990; *Heterocephalus glaber* , O'Riain et al. 1996; *Otus asio* and *O. kennicottii*, Belthoff and Dufty Jr 1998; *Spermophilus beldingi* , Nunes et al. 1998; *Lacerta vivipara* , Meylan et al. 2002; and *Phoenicopterus ruber roseus*, Barbraud et al. 2003). The current study demonstrates that larval body size varies in response to parental habitat quality and supports the hypothesis of plasticity of a dispersal‐related trait in a marine fish.

An alternative way of thinking about the observed plastic response in offspring body size is to consider its consequence on survival. While there are mechanistic reasons why larger offspring may disperse more often or farther (e.g., enhanced walking, swimming, flying), it is also widely supported that larger offspring have greater survival (Roff 1992). For marine fishes, mortality rates are especially high during larval dispersal (Bailey and Houde 1989, Leggett and Deblois 1994, Almany and Webster 2006), and larger body sizes at hatch result in greater survival and higher recruitment rates (e.g., *Salmo salar* , Einum and Fleming 2000; *Neopomacentrus filamentosus* , Vigliola and Meekan 2002; *Symphodus roissali* and *Symphodus ocellatus* , Raventós and Macpherson 2005; *Spratelloides gracilis* , Meekan et al. 2006; *Ocyurus chrysurus* and *Lutjanus synagris* , D'Alessandro et al. 2013; *Sardina pilchardus* , Garrido et al. 2015; reviewed by Miller et al. 1992, Anderson 1988, Houde 1997, Sogard 1997, and Johnson et al. 2014). Survival during the larval stage may be enhanced by large body sizes due to their effect on greater energy reserves (e.g., Gagliano et al. 2007), predator evasion (e.g., Cowan Jr. et al. 1996), and/or ability to catch food (e.g., Dower et al. 2009). Thus, the plasticity of body size might be adaptive for survival, and any consequences for dispersal (e.g., Cowen 2002) might simply be byproducts.

### 4.2 No Evidence of Plasticity in Larval Swimming Speed

We did not find an effect of parental food ration on swimming speed (Ucrit). However, we are cautious with this conclusion because it is difficult to measure a behavioral trait such as Ucrit; it has greater phenotypic variability, and thus it is harder to detect an effect of one predictor variable, such as parental diet. In support of this, it is worth noting that our manipulation influenced both mean larval body size and mean larval Ucrit by 3%, but the standard errors for Ucrit were much greater. This variability may come from our inability to standardize the individual experience of every sampled larva, such as the number of rotifers it ate prior to the swimming flume trial, for example. Additionally, while standard length is a trait that can be measured with high repeatability, performance in a swimming flume could be different every time we measured if we repeated trials for an individual. Unfortunately, we cannot measure Ucrit multiple times because the trial is designed to measure the moment a larva has exhausted its energy and can no longer maintain its position against the current. Any possible subsequent trials would be inaccurate, measuring an already exhausted individual. Additionally, a nonsignificant result from determining a predictor of Ucrit of Day 0 larvae is consistent with similar studies. Schlatter et al. (2022) found only equivocal evidence for heritability of Ucrit, despite finding unequivocal evidence for heritability of larval body size. Also, Fuiman and Cowan Jr (2003) showed no repeatability in behavioral assays of larval swimming, highlighting the difficulty in determining predictors of the variation.

Given the significance of Ucrit in predicting larval dispersal distance (Burgess et al. 2022), it is worth considering our result of a significant effect of parental environment on larval body size and prior evidence that body size predicts Ucrit. Through ontogeny of the larval stage, larger body sizes are correlated with faster swimming speeds (Fisher et al. 2005; Majoris et al. 2019). Importantly, body size is positively related to swimming speed even at early developmental stages in *Amphiprion* larvae (Bellwood and Fisher 2001) and other fishes (e.g., Fisher and Hogan 2007; Downie et al. 2021). Previous studies have shown that larvae with larger body size at hatch have faster growth (Vigliola and Meekan 2002; D'Alessandro et al. 2013), suggesting larger larvae at hatch may maintain their relatively larger body sizes through ontogeny because of faster growth compared to larvae with smaller body size‐at‐hatch. To put our finding of a 0.12 mm difference in larval body size between treatments into context, larvae of a closely related species, *Amphiprion ocellaris* , grow about 0.2 mm per day during the first week post hatch (Roux et al. 2019). In sum, larger offspring at hatch may also then maintain greater swimming speeds through the larval dispersal stage than smaller offspring and therefore may have greater propensities to disperse greater distances.

### 4.3 Future Indirect Test of the Dispersal Plasticity Hypothesis Using Otoliths

Otolith analyses provide a convenient history of a fish's age, growth, and, importantly, body size at hatch from back calculations (e.g., Meekan et al. 1998; D'Alessandro et al. 2013; Garrido et al. 2015). We found a significant effect of parental food ration on otolith core size. Curiously, we found that otolith core major diameter is smaller in offspring from parents during the “low ration” treatment, even though those offspring were larger than offspring from parents in the “high ration” treatment. Although unexpected, this effect of poor parental environments resulting in smaller otolith core size (sometimes referred to as “hatch‐check”) is seen elsewhere (e.g., cod, Grønkjær and Schytte 1999). Taken together, our contradictory result of larger offspring body size and smaller otolith core sizes may reveal that parents do not just produce bigger larvae in response to poor environments; rather, they produce larvae with a specific suite of dispersal‐related traits (i.e., dispersal syndrome) that may be matched to environmental conditions (Sih et al. 2012; Cote et al. 2022). Importantly, one could use otoliths collected from specimens in the field to perform an indirect test of the dispersal plasticity hypothesis. For example, one could test whether settled larvae, or “recruits,” with small, medium, or large otolith cores have different dispersal distance distributions estimated from parentage analysis.

### 4.4 Future Direct Tests of the Dispersal Plasticity Hypothesis

In this study, we found plasticity of a dispersal‐related trait in response to variation in the parental environment. However, to demonstrate dispersal plasticity in *A. percula* , we still need to conduct a direct test of the plasticity of larval dispersal distance. Given that there is positive spatial autocorrelation of habitat quality in the wild (anemone size, female size, clutch size; Francis et al. 2024; Marrot et al. 2025), selection might favor parents in poor habitats that produce larvae that disperse out of those habitats and parents from good habitats that produce larvae that remain in those habitats. To test this hypothesis observationally, one would need to test whether parents in good habitats (large females living in large anemones and producing large egg clutches) have a greater fraction of their offspring dispersing short distances and a smaller fraction of their offspring dispersing long distances, relative to parents in poor habitats (small females living in small anemones producing small egg clutches). This requires measuring body size, habitat quality, and reproduction of breeding pairs (e.g., Barbasch et al. 2020) and the dispersal of larvae using parentage analysis (e.g., Almany et al. 2017) at the same time and place. To test this hypothesis experimentally, one could manipulate the parental habitat quality, either by supplementing food availability (e.g., Barbasch et al. 2020) or by moving breeding pairs between high‐quality and low‐quality habitats (e.g., Branconi et al. 2020) and measuring the response in offspring' dispersal distance.

### 4.5 Broader Implications for Marine Fish Population Dynamics

While parental diet explains 16% of the variation in larval size in this population, Schlatter et al. (2022) found additive genetic variance explains 20% of the variation in larval size in this population. Together, we explain a large portion of the variation in this important dispersal‐related trait, suggesting body size may be described by genetic and environmental interactions. In terms of marine fish dispersal patterns as a result of variation in larval body sizes, this trait‐based approach has the potential to provide a more predictive framework for metapopulation ecology (De Bie et al. 2012). How much of the variation in intraspecific dispersal distance we can explain is an open question. Recent studies have shown that marine fish dispersal kernels vary year to year quite substantially (Almany et al. 2017; Catalano et al. 2021). However, within any given year, there is still substantial variation yet to be explained. The results of this study suggest the dispersal plasticity hypothesis has the potential to explain some of this four‐to‐five‐orders‐of‐magnitude variation in individual dispersal distance.

Incorporating plasticity of body size and other dispersal‐related traits in our investigations of marine fish dispersal patterns may enhance fishery management (Kritzer and Sale 2004; Harrison et al. 2012; Almany et al. 2013) and reserve design (Botsford et al. 2009; Moffitt et al. 2011; Di Franco et al. 2012; Anadón et al. 2013; Green et al. 2015; Abesamis et al. 2017). Variation in dispersal distances in response to parental habitat quality may help to explain why certain reef fish populations collapse. Fishing and other pressures may be adjusting the dispersal strategies of the remaining fish, exacerbating the effect on population levels if offspring disperse elsewhere rather than recruit to the local population. If habitat quality is improved, then dispersal plasticity may drive larvae to remain within the population, re‐establishing healthy population levels. In sum, dispersal plasticity may be a currently overlooked process that could explain fisheries collapse and reserve recovery by influencing the variation we observe in marine fish larval dispersal distances.

## Author Contributions

**Robin K. Francis:** conceptualization (equal), data curation (lead), formal analysis (lead), investigation (lead), methodology (lead), visualization (lead), writing – original draft (lead), writing – review and editing (equal). **Kurt G. Castro:** data curation (supporting), investigation (supporting), methodology (supporting), writing – review and editing (supporting). **Sadie Thompson:** conceptualization (supporting), methodology (supporting). **Isabela Trumble:** project administration (supporting), resources (supporting), supervision (supporting), writing – review and editing (supporting). **John E. Majoris:** conceptualization (supporting), investigation (supporting), methodology (supporting), writing – review and editing (equal). **Peter M. Buston:** conceptualization (equal), formal analysis (equal), funding acquisition (equal), investigation (equal), methodology (equal), project administration (lead), resources (lead), supervision (lead), writing – original draft (supporting), writing – review and editing (equal).

## Conflicts of Interest

The authors declare no conflicts of interest.

## Supporting information


**Data S1:** ece371967‐sup‐0001‐Supinfo01.docx.

## Data Availability

Data and R scripts are publicly available on GitHub at https://github.com/robinkellyfrancis/AperculaDispPlast and on Dryad https://doi.org/10.5061/dryad.bg79cnpkn.

## Appendix A

### Larval Body Morphology Metrics

Larval body length, body depth, muscle area, and propulsive fin area were all significantly and positively correlated to one another (Figure A1).

### Effect of Treatment on Body Morphology Metrics

Results from our mixed models show that treatment also had a significant effect on body depth, muscle area, and fin area (Table A1). Larvae from parents on a low ration were larger in all metrics of larval morphology (Table A1; Figure A2). The results of our final models for each body morphology metric are summarized below (Table A1). For length, which is reported in the paper, neither round nor the interaction between treatment and round were significant predictors and were removed from the final model. For body depth, treatment and the interaction between round and treatment were significant predictors and round was a marginally significant predictor. For muscle area, neither round nor the interaction between treatment and round were significant predictors and were removed from the final model, and treatment was a significant predictor. For propulsive fin area, the interaction between treatment and round was not a significant predictor and was removed from the final model. Both treatment and round were significant predictors.

**TABLE A1:** Summary of the effect of treatment on all body morphology metrics. Result summary of mixed models for the effect of treatment, round, and the interaction between treatment and round on larval length, body depth, muscle area, and propulsive fin area. Non‐significant variables with *p* > 0.1 were removed from the final model (n.s.).

	Variable	Regression coefficient	*t*	Degrees of freedom	*p*	Adj. *R* ^2^
Length	Treatment	0.117 mm	11.23	492.0	< 0.0001	0.164
	Round	n.s.	n.s.	n.s.	n.s.	
	Interaction	n.s.	n.s.	n.s.	n.s.	
Body depth	Treatment	0.038 mm	3.522	17.7	0.0025t	0.113
	Round	0.021 mm	−2.479	17.7	0.025	
	Interaction	−0.05 mm	1.986	17.7	0.07	
Muscle area	Treatment	0.059 mm^2^	9.066	492.0	< 0.0001	0.117
	Round	n.s.	n.s.	n.s.	n.s.	
	Interaction	n.s.	n.s.	n.s.	n.s.	
Fin area	Treatment	0.026 mm^2^	3.345	491.0	0.0009	0.074
	Round	0.043 mm^2^	5.482	491.0	< 0.0001	
	Interaction	n.s.	n.s.	n.s.	n.s.	

### Larval Otolith Core Metrics

Larval otolith major diameter, minor diameter, measured area, and calculated area were all significantly and positively correlated to one another (Figure A3).

### Effect of Treatment on Otolith Metrics

Results from our mixed models show that treatment had a significant effect on the major diameter and a marginally significant effect on the minor diameter, measured area and calculated area (Table A2). Otoliths were all‐around smaller when parents were on the “low ration” (Table A2; Figure A4). The effect of treatment from our final models for each otolith metric are summarized below (Table A2). For major diameter, which is reported in the paper, neither round nor the interaction between treatment and round were significant predictors and were removed from the final model. Treatment had a significant effect. For minor diameter, the interaction between treatment and round was not a significant predicator, and was removed from the final model. Treatment had a marginally significant effect and round had a significant effect. For the measured area, neither round nor the interaction between treatment and round were significant predictors and were removed from the final model. Treatment had a marginally significant effect. For the calculated area, the interaction between treatment and round was not a significant predictor and was removed from the final model. Both treatment and round had a marginally significant effect.

**TABLE A2:** Mixed model result summary for otolith metrics. Effect of low ration treatment on larval otolith major diameter, minor diameter, measured area, and calculated area.

	Variable	Regression coefficient	*t*	Degrees of freedom	*p*	Adj. *R* ^2^
Major diameter	Treatment	−1.3009 μm	−2.22	399	0.027	0.009
	Round	n.s.	n.s.	n.s.	n.s.	
	Interaction	n.s.	n.s.	n.s.	n.s.	
Minor diameter	Treatment	−0.9416 μm	−1.965	398	0.0501	0.019
	Round	1.002 μm	2.091	398	0.0371	
	Interaction	n.s.	n.s.	n.s.	n.s.	
Measured area	Treatment	−11.467 μm^2^	−1.705	399	0.0891	0.005
	Round	n.s.	n.s.	n.s.	n.s.	
	Interaction	n.s.	n.s.	n.s.	n.s.	
Calculated area	Treatment	−13.132 μm^2^	−1.879	398	0.0609	0.014
	Round	13.132	1.799	398	0.0728	
	Interaction	n.s.	n.s.	n.s.	n.s.	
