# Supplementary material for: Plasticity of Dispersal‐Related Larval Traits in the Clown Anemonefish Amphiprion percula
Source: Ecol Evol. 2025 Aug 15;15(8):e71967. doi: 10.1002/ece3.71967 (PMC12356648; doi:10.1002/ece3.71967)
Supplement: Supplementary file 1 — Data S1: ece371967‐sup‐0001‐Supinfo01.docx. [file ECE3-15-e71967-s001.docx]

**Appendix**

*Larval Body Morphology Metrics*

Larval body length, body depth, muscle area, and propulsive fin area were all significantly and positively correlated to one another (Appendix Figure A1).


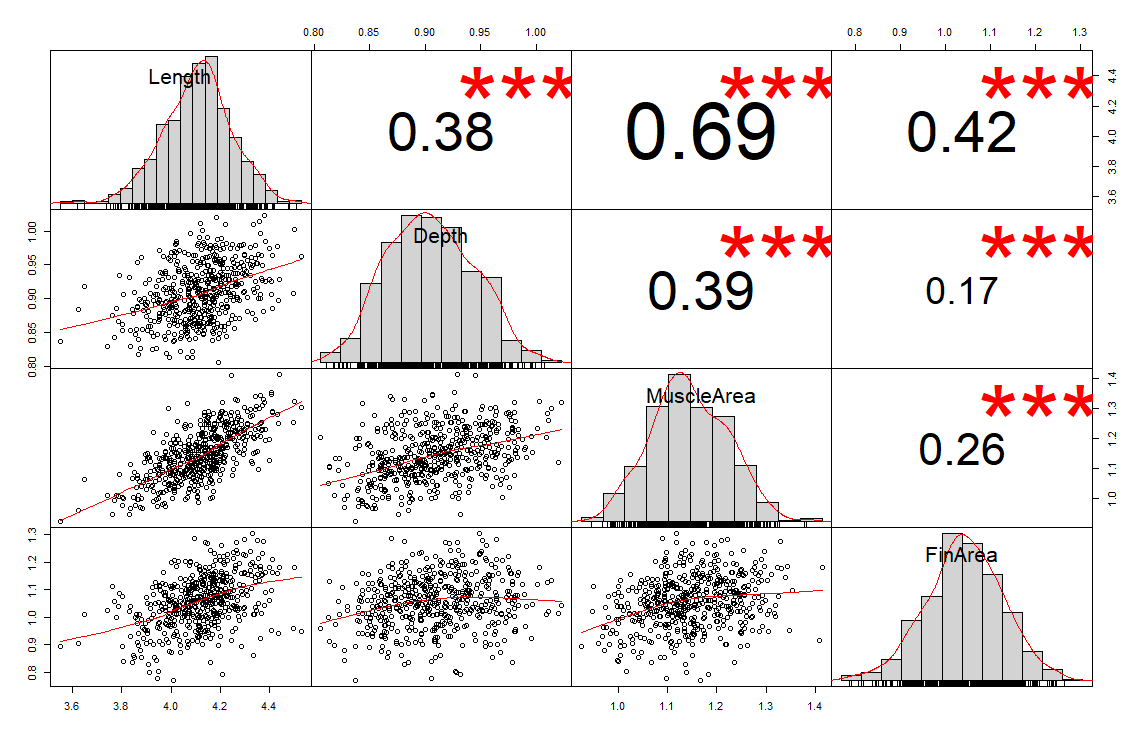

**Appendix Figure A1. Correlogram of Larval Morphology.** Each row is a body morphology metric, from the top to bottom: larval body length (Length), body depth (Depth), muscle area (Muscle Area), and propulsive fin area (Fin Area). The frequency distribution of each variable is shown on the diagonal, with the density curve in red. On the bottom of the diagonal is the bivariate scatterplot with the fitted line in red. On the top of the diagonal is the correlation coefficient and associated significance level (***; p-value < 0.001). The relative font size signifies value of the correlation.

*Effect of Treatment on* *Body Morphology Metrics*

Results from our mixed models show that treatment also had a significant effect on body depth, muscle area, and fin area (Appendix Table A1). Larvae from parents on a low ration were larger in all metrics of larval morphology (Appendix Table A1; Appendix Figure A2). The results of our final models for each body morphology metric are summarized below (Appendix Table A1). For length, which is reported in the paper, neither round nor the interaction between treatment and round were significant predictors and were removed from the final model. For body depth, treatment and the interaction between round and treatment were significant predictors and round was a marginally significant predictor. For muscle area, neither round nor the interaction between treatment and round were significant predictors and were removed from the final model, and treatment was a significant predictor. For propulsive fin area, the interaction between treatment and round was not a significant predictor and was removed from the final model. Both treatment and round were significant predictors.

**Appendix Table A1. Summary of the Effect of Treatment on All Body Morphology Metrics.** Result summary of mixed models for the effect of treatment, round, and the interaction between treatment and round on larval length, body depth, muscle area, and propulsive fin area. Non-significant variables with p > 0.1 were removed from the final model (n.s.).

|  | **Variable** | **Regression Coefficient** | **t-value** | **Degrees of Freedom** | **p-value** | **Adj. R^2^** |
| --- | --- | --- | --- | --- | --- | --- |
| **Length** | Treatment | 0.117 mm | 11.23 | 492.0 | <0.0001 | 0.164 |
|  | Round | *n.s.* | *n.s.* | *n.s.* | *n.s.* |  |
|  | Interaction | *n.s.* | *n.s.* | *n.s.* | *n.s.* |  |
| **Body Depth** | Treatment | 0.038 mm | 3.522 | 17.7 | 0.0025t | 0.113 |
|  | Round | 0.021 mm | -2.479 | 17.7 | 0.025 |  |
|  | Interaction | -0.05 mm | 1.986 | 17.7 | 0.07 |  |
| **Muscle Area** | Treatment | 0.059 mm^2^ | 9.066 | 492.0 | <0.0001 | 0.117 |
|  | Round | *n.s.* | *n.s.* | *n.s.* | *n.s.* |  |
|  | Interaction | *n.s.* | *n.s.* | *n.s.* | *n.s.* |  |
| **Fin Area** | Treatment | 0.026 mm^2^ | 3.345 | 491.0 | 0.0009 | 0.074 |
|  | Round | 0.043 mm^2^ | 5.482 | 491.0 | <0.0001 |  |
|  | Interaction | *n.s.* | *n.s.* | *n.s.* | *n.s.* |  |

| 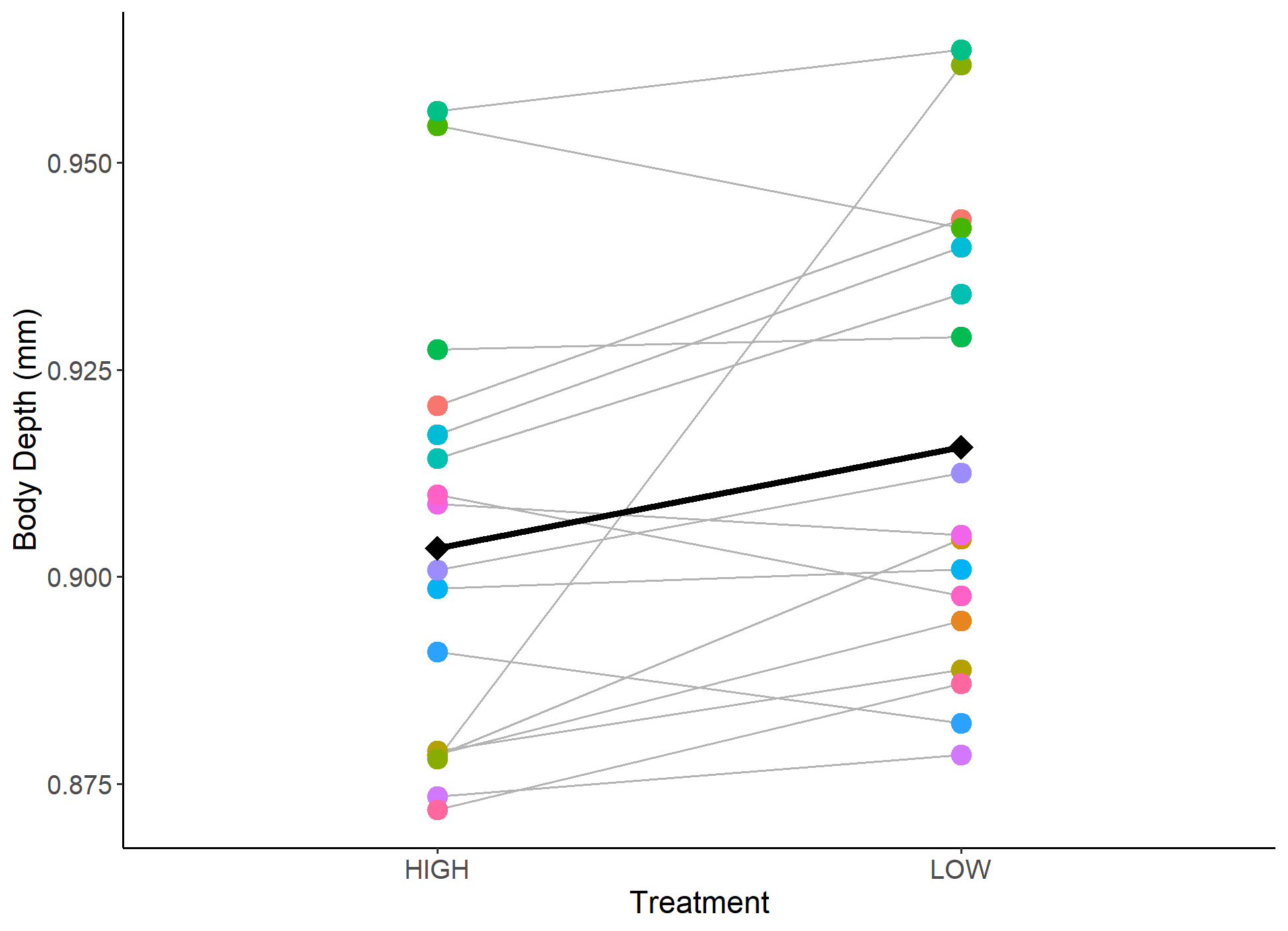(a) | (b) 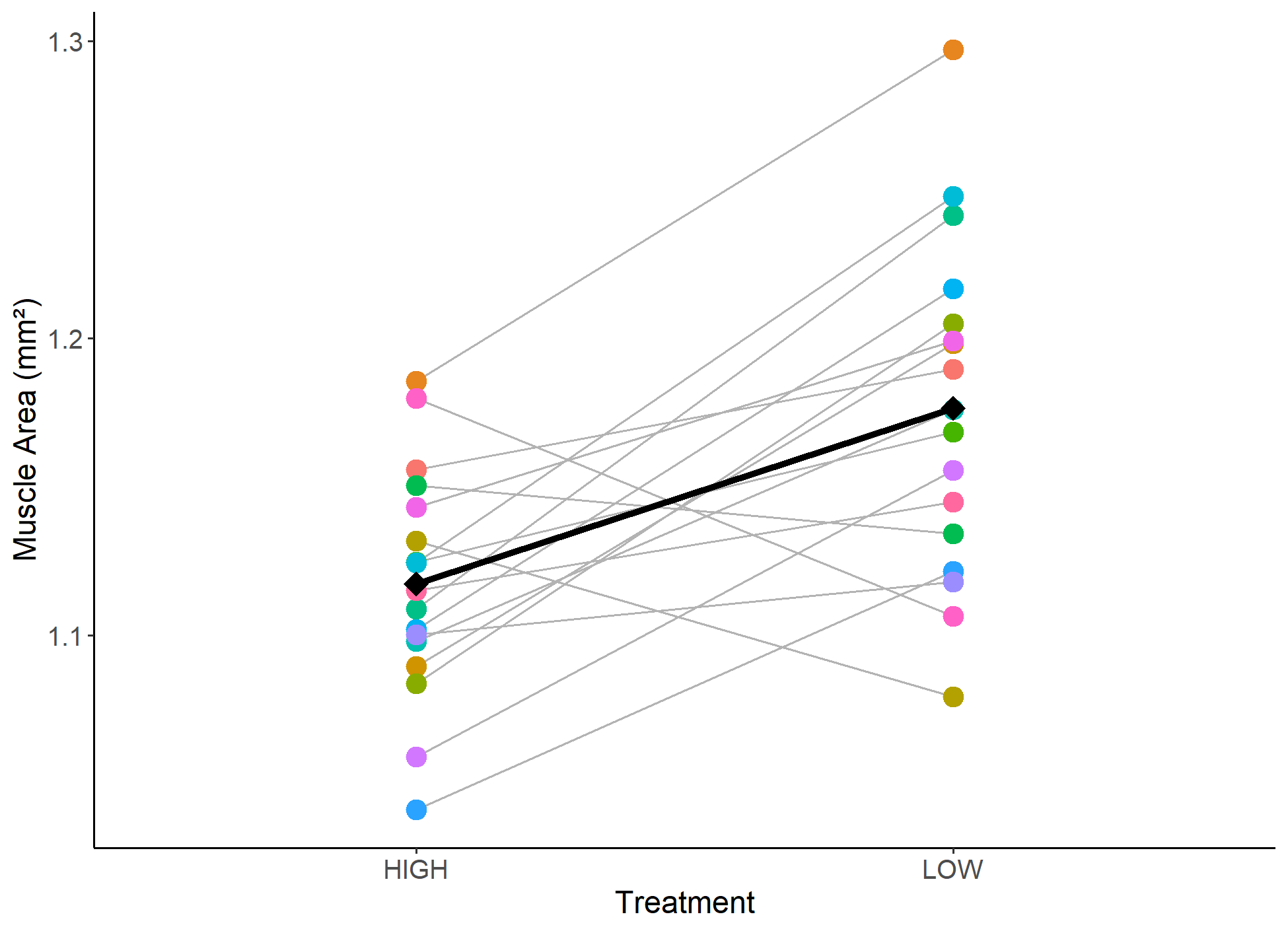 | (c) 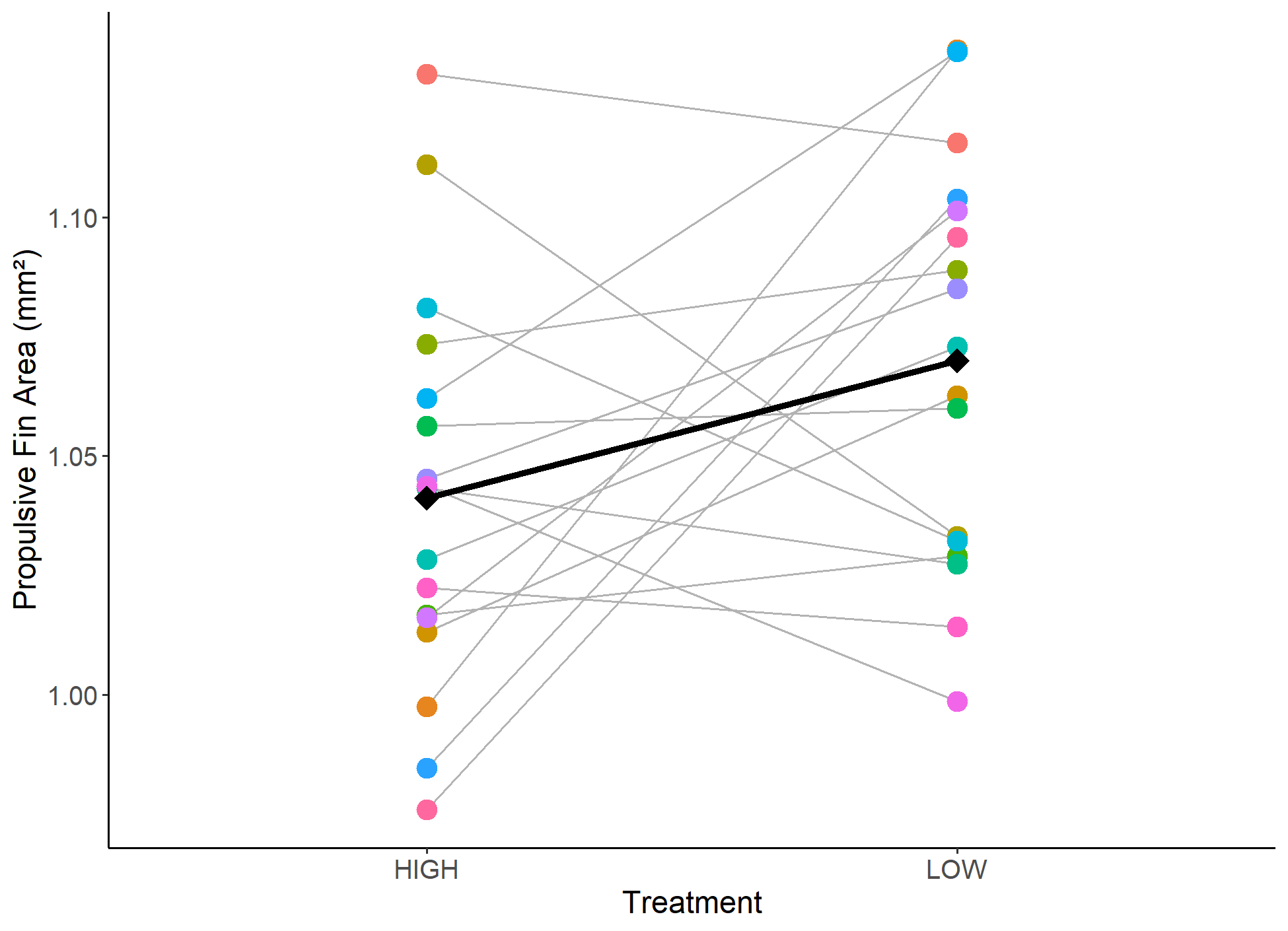 |
| --- | --- | --- |

**Appendix Figure A2. Individual Pair Reaction Norms for (a) body depth, (b) muscle area, and (c) fin area.** Each point represents the average metric value for each clutch per pair, per treatment. Points are individually colored by the unique ‘tank ID’ of each parental pair and may be stacked on top of one another. The grey fitted line connects the pair’s measured values and represents the response to the two treatments. Diamonds represent the mean value for all pairs per treatment and the black line represents the mean response.

*Larval Otolith Core Metrics*

Larval otolith major diameter, minor diameter, measured area, and calculated area were all significantly and positively correlated to one another (Appendix Figure A3).


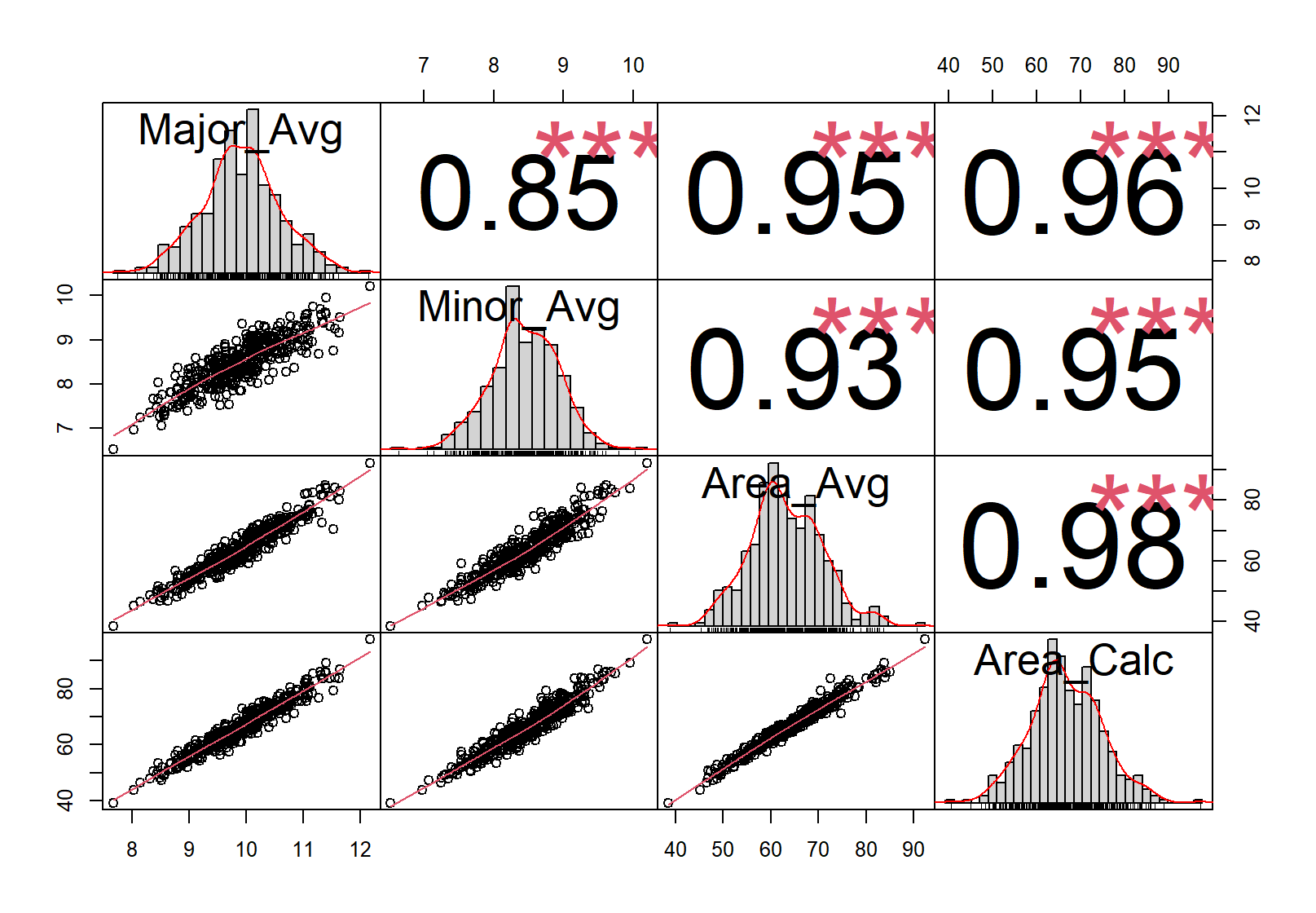


**Appendix Figure A3.** **Correlogram of Larval Otolith Metrics.** Each row is an otolith metric, from the top to bottom: major diameter (Major Avg), minor diameter (Minor Avg), measured area (Area Avg) and calculated area (Area Calc). The frequency distribution of each variable is shown on the diagonal, with the density curve in red. On the bottom of the diagonal is the bivariate scatterplot with the fitted line in red. On the top of the diagonal is the correlation coefficient and associated significance level (***; p-value < 0.001). The font size signifies value of the correlation.

*Effect of Treatment on* *Otolith Metrics*

Results from our mixed models show that treatment had a significant effect on the major diameter and a marginally significant effect on the minor diameter, measured area and calculated area (Appendix Table A2). Otoliths were all-around smaller when parents were on the ‘low ration’ (Appendix Table A2; Appendix Figure A4). The effect of treatment from our final models for each otolith metric are summarized below (Appendix Table A2). For major diameter, which is reported in the paper, neither round nor the interaction between treatment and round were significant predictors and were removed from the final model. Treatment had a significant effect. For minor diameter, the interaction between treatment and round was not a significant predicator, and was removed from the final model. Treatment had a marginally significant effect and round had a significant effect. For the measured area, neither round nor the interaction between treatment and round were significant predictors and were removed from the final model. Treatment had a marginally significant effect. For the calculated area, the interaction between treatment and round was not a significant predictor and was removed from the final model. Both treatment and round had a marginally significant effect.

**Appendix Table A2. Mixed Model Result Summary for Otolith Metrics.** Effect of low ration treatment on larval otolith major diameter, minor diameter, measured area, and calculated area.

|  | **Variable** | **Regression Coefficient** | **t-value** | **Degrees of Freedom** | **p-value** | **Adj. R^2^** |
| --- | --- | --- | --- | --- | --- | --- |
| **Major Diameter** | Treatment | - 1.3009 µm | - 2.22 | 399 | 0.027 | 0.009 |
|  | Round | *n.s.* | *n.s.* | *n.s.* | *n.s.* |  |
|  | Interaction | *n.s.* | *n.s.* | *n.s.* | *n.s.* |  |
| **Minor Diameter** | Treatment | - 0.9416 µm | - 1.965 | 398 | 0.0501 | 0.019 |
|  | Round | 1.002 µm | 2.091 | 398 | 0.0371 |  |
|  | Interaction | *n.s.* | *n.s.* | *n.s.* | *n.s.* |  |
| **Measured Area** | Treatment | - 11.467 µm ^2^ | - 1.705 | 399 | 0.0891 | 0.005 |
|  | Round | *n.s.* | *n.s.* | *n.s.* | *n.s.* |  |
|  | Interaction | *n.s.* | *n.s.* | *n.s.* | *n.s.* |  |
| **Calculated Area** | Treatment | - 13.132 µm ^2^ | -1.879 | 398 | 0.0609 | 0.014 |
|  | Round | 13.132 | 1.799 | 398 | 0.0728 |  |
|  | Interaction | *n.s.* | *n.s.* | *n.s.* | *n.s.* |  |

| (a) | (b) | (c) |
| --- | --- | --- |
| 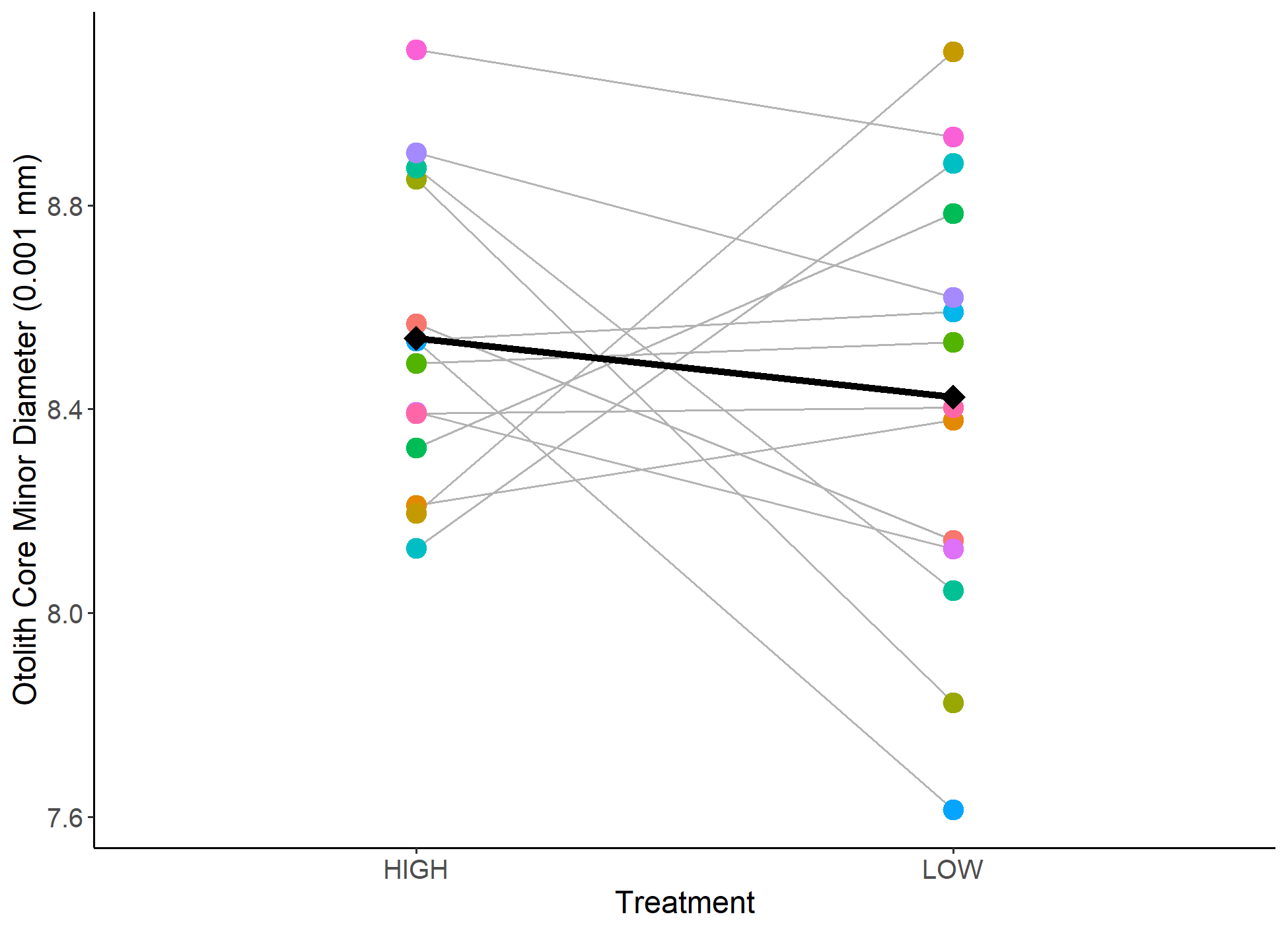 | 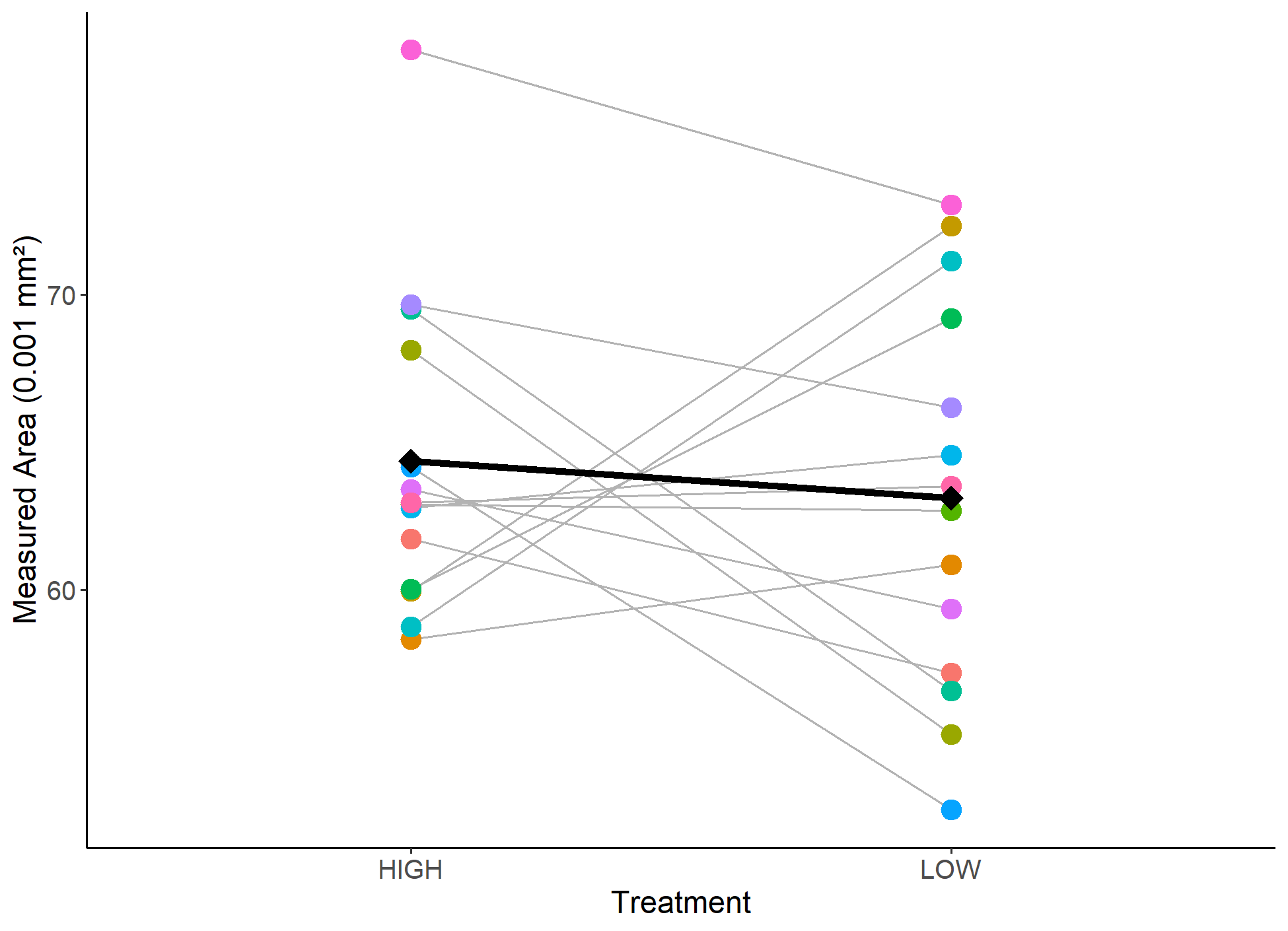 | 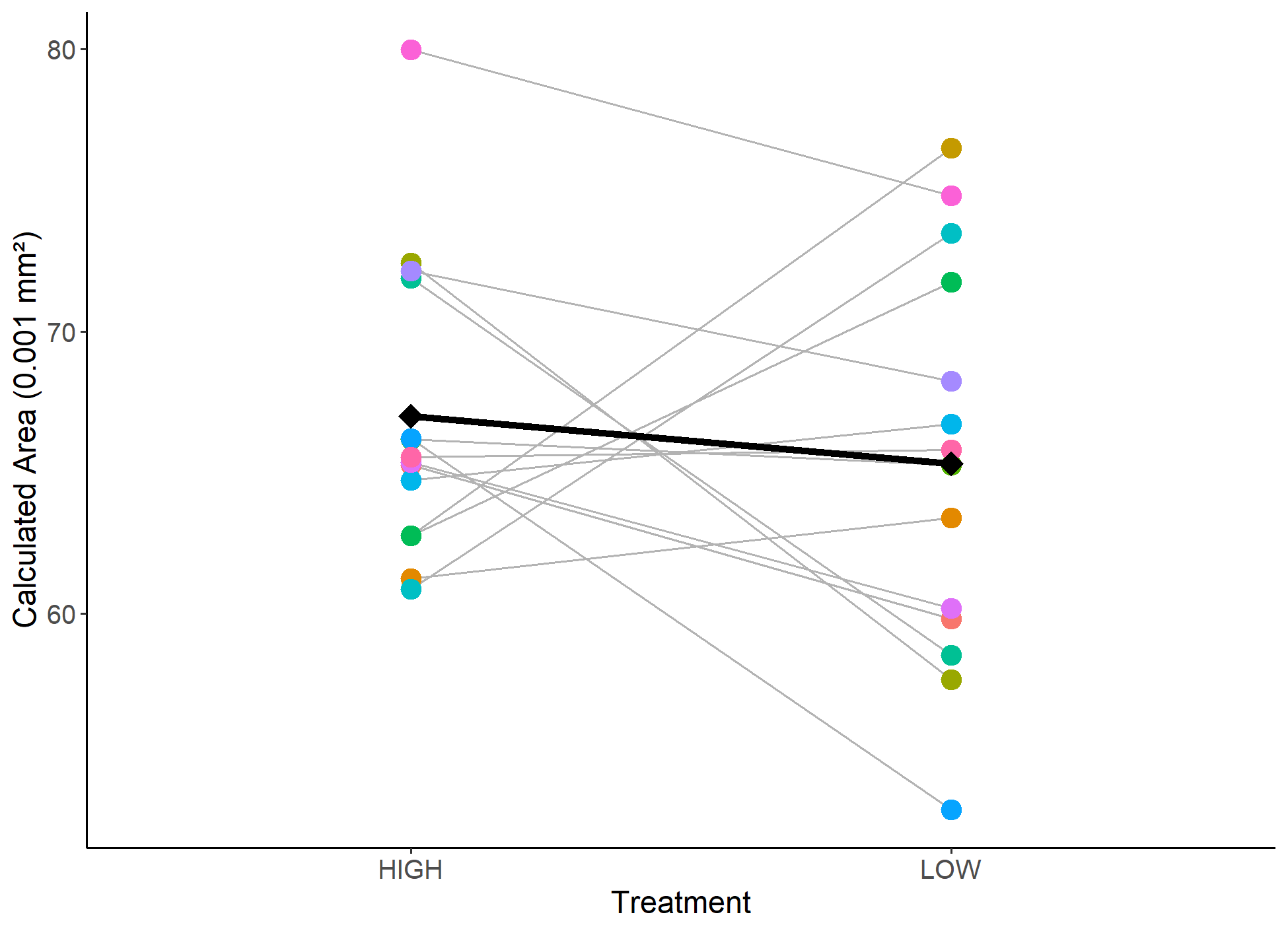 |

**Appendix Figure A4. Individual Pair Reaction Norms for otolith (a) minor diameter, (b) measured area, and (c) calculated area.** Each point represents the average metric value for each clutch per pair, per treatment. Points are individually colored by the unique ‘tank ID’ of each parental pair and may be stacked on top of one another. The grey fitted line connects the pair’s measured values and represents the response to the two treatments. Diamonds represent the mean value for all pairs per treatment and the black line represents the mean response.
